# Hybrid Aortic Arch Repair for Patients Older Than 60 Years in Type A Acute Aortic Dissection

**DOI:** 10.1055/a-2777-5861

**Published:** 2026-01-07

**Authors:** Nianguo Dong, Junwei Liu, Xinling Du, Xionggang Jiang, Long Wu, Hao Hong

**Affiliations:** Department of Cardiovascular Surgery, Union Hospital, Tongji Medical College, Huazhong University of Science and Technology, Wuhan, China

**Keywords:** type A aortic dissection, hybrid aortic arch repair, elderly

## Abstract

**Background:**

The objective of this study was to compare clinical outcomes of total arch replacement (TAR) combined with stented elephant trunk (SET) implantation and hybrid aortic arch repair (HAAR) for type A acute aortic dissection (TA-AAD) in patients older than 60 years.

**Patients and Methods:**

We studied records of patients with TA-AAD older than 60 years in our hospital between January 2016 and December 2018. About 68 patients underwent TAR combined with SET implantation (SET group), and 56 patients underwent HAAR (hybrid group). Outcomes included operative data, postoperative data, and 2 years of follow-up data.

**Results:**

Comparing with the SET group, the hybrid group experienced shorter time on surgery duration ( *p* < 0.001), cardiopulmonary bypass ( *p* < 0.001), aortic cross-clamp ( *p* < 0.001), mechanical ventilation ( *p* < 0.001), ICU stay ( *p* < 0.001), and hospital length of stay ( *p* < 0.001). The hybrid group showed a lower rate of pulmonary infection and renal failure ( *p* = 0.023; *p* = 0.022, respectively). Blood product use was less in the hybrid group ( *p* *<* 0.001). The hybrid group had a trend toward reducing the 30-day mortality rate, stroke, and transient mental dysfunction. The hybrid group had a trend toward improving the 2-year survival rate and reintervention-free rate, but the results did not reach a significant level.

**Conclusion:**

Hybrid procedure could be safely performed in patients older than 60 years with TA-AAD. This procedure may be associated with encouraging surgical results and promising outcomes in the early and mid-term.

## Introduction

Type A acute aortic dissection (TA-AAD) involving the aortic arch remains a lethal condition. The appropriate approach for these patients remains controversial. Total arch replacement (TAR) combined with stented elephant trunk (SET) implantation is associated with encouraging surgical results and promising outcomes. 1 2 But TAR + SET increases the risk for postoperative death, especially in elderly patients. Recently, the elderly are more prone to cardiovascular diseases due to lifestyle upgrades. 3 Moreover, previous studies reported that age was an independent predictor of long-term mortality after TAR surgery. 4 The resulting weakened buffering capacity of vital organs, defensive ability, and adaptability to surgery, as well as postoperative dysfunction of organs, easily induces complications. 5 Given the organ dysfunction of elderly patients, the incidence of postoperative complications and mortality rate is often high. 6

As an ongoing process of innovation, the endovascular technique inspired the development of an alternative treatment for TA-AAD, namely hybrid aortic arch repair (HAAR) technique. This technique comprises an open portion of the aortic root, ascending aortic replacement, and revascularization of supra-arch branches, and an endovascular portion of the exclusion of the transversal aortic arch from the stent graft. However, little is known about the actual application of the HAAR procedure in patients older than 60 years with TA-AAD. Furthermore, contemporary literature comparing the TAR + SET and the HAAR procedures in terms of early and mid-term clinical outcomes is limited. In this study, we analyzed the clinical data from the TAR + SET and the HAAR procedures performed in patients older than 60 years with TA-AAD in our hospital.

## Patients and Methods

We retrospectively studied the records of patients older than 60 years with TA-AAD. These patients underwent surgical treatment in our hospital between January 2016 and December 2018. The inclusion criteria were as listed: (1) All the surgeries were conducted during the acute phase of the disease (<48 hours); (2) the indications for extensive repair included an intimal tear located in the ascending aorta, aortic arch, or descending thoracic aorta (DTA), and dissection extending into ascending aorta, aortic arch, and DTA. We divided the patients into two groups according to surgical procedure: TAR + SET ( *n* = 68) and HAAR ( *n* = 56). We excluded patients undergoing concomitant valve or coronary operations, past cardiovascular surgery, Marfan syndrome, acute myocardial infarction, and acute left heart failure to minimize discrepancies in disease extent between the two groups. All the operations were performed by one team of aortic surgeons. The study protocol was approved by the Institutional Review Board of our hospital. Informed consent was obtained from each patient involved in this study.

## Surgical Techniques

### Total Arch Replacement +Stented Elephant Trunk Group

All procedures were performed by a median sternotomy and total cardiopulmonary bypass (CPB) with selective cerebral perfusion (SCP) ( Fig. 1A ). Cannulation of the right axillary artery was used for CPB and SCP. Aortic root operation (see Table 1 ) was performed during cooling after CPB. Deep hypothermic circulatory arrest (DHCA) was initiated at a nasopharyngeal temperature of 18 to 20 °C. 2 Unilateral SCP was started through the right axillary artery after the brachiocephalic arteries were cross-clamped and the brain was perfused. The aorta was opened longitudinally between the left common carotid artery and the left subclavian artery (LSA), and the SET (MicroPort Medical Co. Ltd., Shanghai, China) was inserted into the true lumen of DTA. Subsequently, the proximal end of the SET and the aortic wall were anastomosed together to the distal end of a 4-branch prosthetic graft (Boston Scientific Inc., Boston, MA). Antegrade systemic perfusion was then resumed through the perfusion limb of the 4-branch prosthetic graft, followed by rewarming. The sequence of anastomosis to the 4-branch prosthetic graft was the left common carotid artery, proximal aortic stump, innominate artery, and LSA. After the anastomosis to the left common carotid artery was accomplished, SCP was discontinued, CPB gradually resumed to normal flow, and rewarming started. 2 After the patient was weaned off from CPB and wound hemostasis was achieved, drainage tubes were placed, and the chest was closed in routine.

**Fig. 1:**
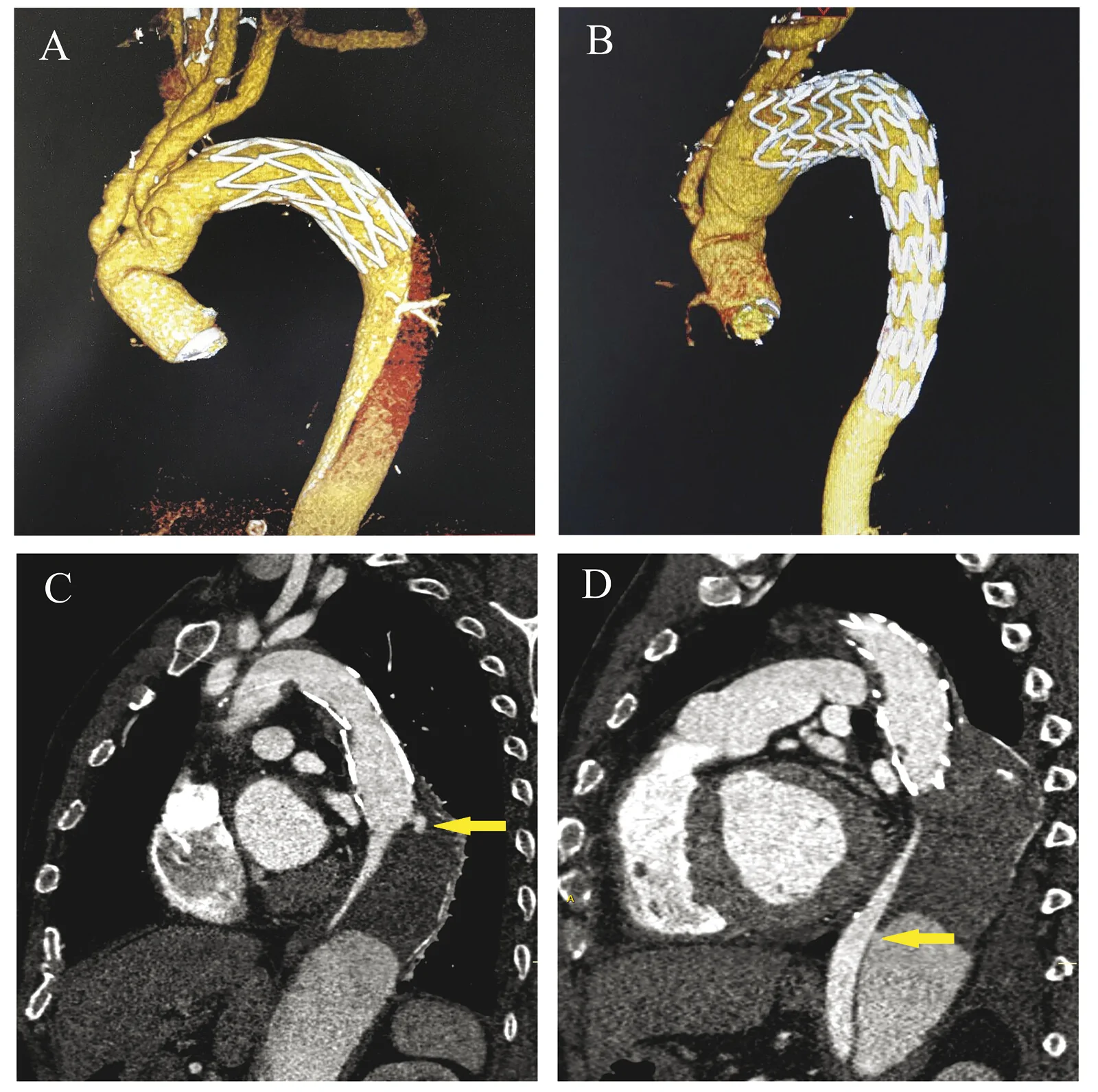
Postoperative CTA. ( **A** ) The TAR + SET technique. ( **B** ) The HAAR technique. ( **C** ) New entry of dissection caused by SET in the TAR + SET group (yellow arrow). ( **D** ) Original entry of dissection in the DTA in the TAR + SET group (yellow arrow). CTA, computed tomography angiography; DTA, descending thoracic aorta; HAAR, hybrid aortic arch repair; SET, stented elephant trunk; TAR, total arch replacement.

**Table 1:** Operative data

	TAR + SET ( *n* = 68)	HAAR ( *n* = 56)	*p* -Value
Aortic root procedure			
Bentall's	11 (16.2%)	12 (21.4%)	0.454
Ascending aortic replacement	57 (83.8%)	44 (78.6%)	0.454
CPB time (minutes)	187.0 ± 20.4	131.9 ± 16.3	<0.001
Cross-clamp time (minutes)	125.6 ± 15.8	78.1 ± 13.0	<0.001
DHCA time (minutes)	27.1 ± 2.4	–	–
Surgery duration (minutes)	403.6 ± 63.0	358.0 ± 65.6	<0.001

Abbreviations: CPB, cardiopulmonary bypass; DHCA, deep hypothermic circulatory arrest; HAAR, hybrid aortic arch repair; SET, stented elephant trunk; TAR, total arch replacement.

### Hybrid Aortic Arch Repair

The procedure was performed under moderate hypothermic CPB in a hybrid endosuite equipped with a fixed C-arm fluoroscopy system (AXIOM Artis FA; Siemens, Malvern, PA) ( Fig. 1B ). Nasopharyngeal temperature was lowered to 28 °C. The right axillary and the femoral arteries were initially separated. After a median sternotomy, CPB was established by arterial cannulation in the right axillary and femoral artery. Meanwhile, a two-stage cannula (Medtronic, Minneapolis, MN) was inserted into the right atrium for venous drainage. The ascending aorta was cross-clamped between the orifice of the innominate artery and the left common carotid artery. After the ascending aorta was opened longitudinally, cold blood cardioplegia was administered directly into the coronary ostia for myocardial protection. The aortic root was carefully inspected, and a suitable operation was performed. The aortic arch was transected proximal to the left common carotid artery. The distal dissected aortic arch stump was reinforced by a pericardium patch strip outside with 4–0 Prolene interrupted stitches. The distal end of the 4-branch prosthetic graft was then sutured end-to-end to the aortic arch stump, proximal to the left common carotid artery. Next, the proximal segment of the 4-branch prosthetic graft was anastomosed to repair the ascending aorta, or the proximal end of the 4-branch prosthetic graft was anastomosed to the distal end of the mechanical valved graft (St. Jude Medical, Inc., St. Paul, MN) that was used to replace the aortic root. Finally, the aortic clamp was removed, and rewarming was initiated. The supra-arch branches were transposed to the limbs of the 4-branch prosthetic graft on the pump. Each branch vessel takeoff was ligated. After the patient was weaned off from CPB and wound hemostasis was achieved, a half dose of protamine was administered for heparin neutralization.

The endovascular portion was processed. The femoral artery was used as the access site for stent delivery. The size of the stent graft was determined by the presutured 4-branch prosthetic graft that was approximately 15% oversized in diameter. The silver clips were marked at the uppermost opening limb of the 4-branch prosthetic graft. The proximal stent graft was deployed and anchored to the 4-branch prosthetic graft with an overlapping margin of approximately 2 cm to complete the aortic arch repair. Two kinds of commercial stent grafts were used: Valiant (Medtronic Inc., Santa Rosa, CA) and Ankura (LifeTech Scientific Co., Ltd., Shenzhen, China). One stent graft (192–200 mm in length) was adequate. Endoleak or incomplete sealing was examined by angiography.

Clinical data were collected from databases in our hospital. The preoperative data included age, sex (male), body mass index (BMI), left ventricular ejection fraction (LVEF), coronary artery disease (CAD), history of stroke, chronic obstructive pulmonary disease (COPD), smoking, hepatic insufficiency, renal dysfunction, hypertension, and diabetes mellitus. The operative data included time of CPB, aortic cross-clamp, DHCA, and surgery duration. The postoperative data included 30-day mortality rate, cardiac dysfunction, pulmonary infection, hepatic insufficiency, renal failure, stroke, transient mental dysfunction, paraplegia, gastrointestinal dysfunction, wound infection, reoperation for bleeding, endoleaks, blood product use, mechanical ventilation time, ICU stay, and hospital stay. Two years of follow-up data were obtained from outpatient visits or telephone interviews. Follow-up protocols, including total aortic computed tomography angiography (CTA), were scheduled for each patient before discharge, at month 6, and annually thereafter to detect thrombosis of the residual false lumen in the downstream aorta and endoleak.

## Statistical Analyses

The following statistical programs were used for analysis: SPSS 21.0 (IBM, Armonk, NY), R version 3.6.3 (R Foundation for Statistical Computing, Vienna, Austria), and SAS 9.4 software (SAS Institute Inc, Cary, NC). Continuous variables were presented in the form of mean ± standard deviation (SD), or median with interquartile range (IQR), and analyzed using the Student's *t* -test or the Mann–Whitney U test. Categorical variables were analyzed by the χ2 test or the Fisher's exact probability test. The Kaplan–Meier method was used to estimate the survival and reintervention rates. A value of *p* < 0.05 was considered significant.

## Results

### Preoperative Characteristics

The preoperative characteristics of the patients are shown in Table 2 , and the patients' characteristics were comparable between the TAR + SET group and the HAAR group. No statistically significant difference was observed in preoperative characteristics ( *p* > 0.05).

**Table 2:** Baseline characteristics of the study cohort

Characteristic	TAR + SET	HAAR	*p* -Value
Overall cohort ( *n* = 124)	*n* = 68	*n* = 56	
Demographics			
Age (years)	64.2 ± 2.9	65.2 ± 3.2	0.074
Male, *n* (%)	55 (80.9%)	45 (80.4%)	0.941
BMI	24.3 ± 0.5	24.2 ± 0.5	0.130
Clinical history			
LVEF	56.2 ± 4.2	54.8 ± 3.9	0.067
Smoking, *n* (%)	20 (29.4%)	18 (32.1%)	0.743
Presentation			
Acute presentation (<48 hours), *n* (%)	68 (100%)	56 (100%)	–
Mechanical ventilation, *n* (%)	0 (0%)	0 (0%)	–
Resuscitation, *n* (%)	0 (0%)	0 (0%)	–
ECMO preoperatively, *n* (%)	0 (0%)	0 (0%)	–
Comorbidities			
CAD, *n* (%)	8 (11.8%)	6 (10.7%)	0.854
History of stroke, *n* (%)	3 (4.4%)	2 (3.6%)	1.000
COPD, *n* (%)	2 (2.9%)	1 (1.8%)	1.000
Hepatic insufficiency, *n* (%)	4 (5.9%)	5 (8.9%)	0.762
Renal dysfunction, *n* (%)	5 (7.4%)	1 (1.8%)	0.309
Hypertension, *n* (%)	58 (85.3%)	46 (82.1%)	0.635
Diabetes mellitus, *n* (%)	6 (8.8%)	7 (12.5%)	0.506
Acute cardiac tamponade, *n* (%)	0 (0%)	0 (0%)	–
Cerebral ischemia, *n* (%)	0 (0%)	0 (0%)	–
Acute visceral ischemia, *n* (%)	0 (0%)	0 (0%)	–
Lower extremity ischemia, *n* (%)	0 (0%)	0 (0%)	–

Abbreviations: BMI, body mass index; CAD, coronary artery disease; COPD, chronic obstructive pulmonary disease; HAAR, hybrid aortic arch repair; LVEF, left ventricular ejection fraction; SET, stented elephant trunk; TAR, total arch replacement.

### Operative Data

The surgery details are presented in Table 1 . Bentall's procedure and ascending aortic replacement were performed in similar proportions in the two groups (16.2% vs. 21.4%, *p* = 0.454; 83.8% vs. 78.6%, *p* = 0.454, respectively). Compared with patients in the TAR + SET group, the patients in the HAAR group experienced shorter time on surgery duration (403.6 ± 63.0 minutes vs. 358.0 ± 65.6 minutes, *p* < 0.001), CPB (187.0 ± 20.4 minutes vs. 131.9 ± 16.3 minutes, *p* < 0.001), aortic cross-clamp (125.6 ± 15.8 minutes vs. 78.1 ± 13.0 minutes, *p* < 0.001), and no DHCA.

### Early Postoperative Data

Patients in the HAAR group spent shorter time on mechanical ventilation (72 [50–96] hours vs. 24 [12–72] hours, *p* < 0.001), ICU stay (6 [4–11] days vs. 4 [2–6] days, *p* < 0.001), and hospital length of stay (21 [16–30] days vs. 15 [12–20] days, *p* < 0.001). Our results demonstrated that the HAAR group had a trend toward reducing 30-day mortality rate, stroke, and transient mental dysfunction, but the results did not reach a significant level (8.8% vs. 1.8%, *p* = 0.194; 5.9% vs. 0%, *p* = 0.182; 4.4% vs. 0%, *p* = 0.315, respectively). In the TAR + SET group, six patients died. The main causes of death were pulmonary infection ( *n* = 5) and cardiac dysfunction ( *n* = 1). In the HAAR group, one death occurred due to cardiac dysfunction. The HAAR group showed a lower rate of pulmonary infection and renal failure than patients in the TAR + SET group (16.2% vs. 3.6%, *p* = 0.023; 11.8% vs. 0%, *p* = 0.022, respectively). Paraplegia did not occur in either group. The incidence of cardiac dysfunction and hepatic insufficiency for the TAR + SET group were 1.5% and 2.9%, respectively, and 3.6% and 1.8% for the HAAR group, respectively, but the results did not reach a significant level ( *p* = 0.865; *p* = 1.000, respectively). Blood product use was less in the HAAR group than in the TAR + SET group (10.8 [9.0–12.0] vs. 6.5 [5.5–8.0], *p* *<* 0.001). One gastrointestinal dysfunction, three wound infections, and two reoperations for bleeding occurred in the TAR + SET group, but the results did not reach a significant level (1.5% vs. 0%, *p* = 1.000; 4.4% vs. 0%, *p* = 0.315; 2.9% vs. 0%, *p* = 0.501, respectively). No endoleak was observed in either group ( Table 3 ).

**Table 3:** Early outcomes

	TAR + SET ( *n* = 68)	HAAR ( *n* = 56)	*p* -Value
30-day mortality rate	6 (8.8%)	1 (1.8%)	0.194
Cardiac dysfunction	1 (1.5%)	2 (3.6%)	0.865
Pulmonary infection	11 (16.2%)	2(3.6%)	0.023
Hepatic insufficiency	2 (2.9%)	1 (1.8%)	1.000
Renal failure	8 (11.8%)	0 (0%)	0.022
Stroke	4 (5.9%)	0 (0%)	0.182
Transient mental dysfunction	3 (4.4%)	0 (0%)	0.315
Paraplegia	0	0	–
Cerebrospinal fluid drainage	0	0	–
Gastrointestinal dysfunction	1 (1.5%)	0 (0%)	1.000
Wound infection	3 (4.4%)	0 (0%)	0.315
Reoperation for bleeding	2 (2.9%)	0 (0%)	0.501
Rupture events	0	0	–
Endoleaks	0	0	–
Blood product use	10.8 (9.0–12.0)	6.5 (5.5–8.0)	<0.001
Mechanical ventilation time	72 (50–96)	24(12–72)	<0.001
ICU stay	6 (4–11)	4(2–6)	<0.001
Hospital stay	21(16–30)	15 (12–20)	<0.001

Abbreviations: HAAR, hybrid aortic arch repair; SET, stented elephant trunk; TAR, total arch replacement.

### Midterm Survival and Reintervention

We followed up 62 patients in the TAR + SET group and 55 patients in the HAAR group. The mean follow-up period was 22.5 ± 5.7 months. The 2-year survival rate was 94.8% in the TAR + SET group and 98.2% in the HAAR group ( Fig. 2A ). The reintervention-free rate at 2 years was 88.7% in the TAR + SET group and 95.4% in the HAAR group ( Fig. 2B ), but the results did not reach a significant level ( *p* = 0.385 and *p* = 0.306, respectively). The causes of death were attributed mainly to complications related to pulmonary infection ( *n* = 2 in the TAR + SET group; *n* = 1 in the HAAR group, respectively) and cerebrovascular accidents ( *n* = 1 in the TAR + SET group). The causes of reintervention were that new entry of dissection caused by the distal end of SET in DTA in the TAR + SET group ( *n* = 3) during follow-up after discharge ( Fig. 1C ). Or original entry of dissection in the DTA in the TAR + SET group ( *n* = 2) was not covered and sealed by the stented elephant trunk in the first surgery ( Fig. 1D ). These patients underwent endovascular intervention during follow-up. Two patients underwent reoperation of aortic root replacement in the HAAR group.

**Fig. 2:**
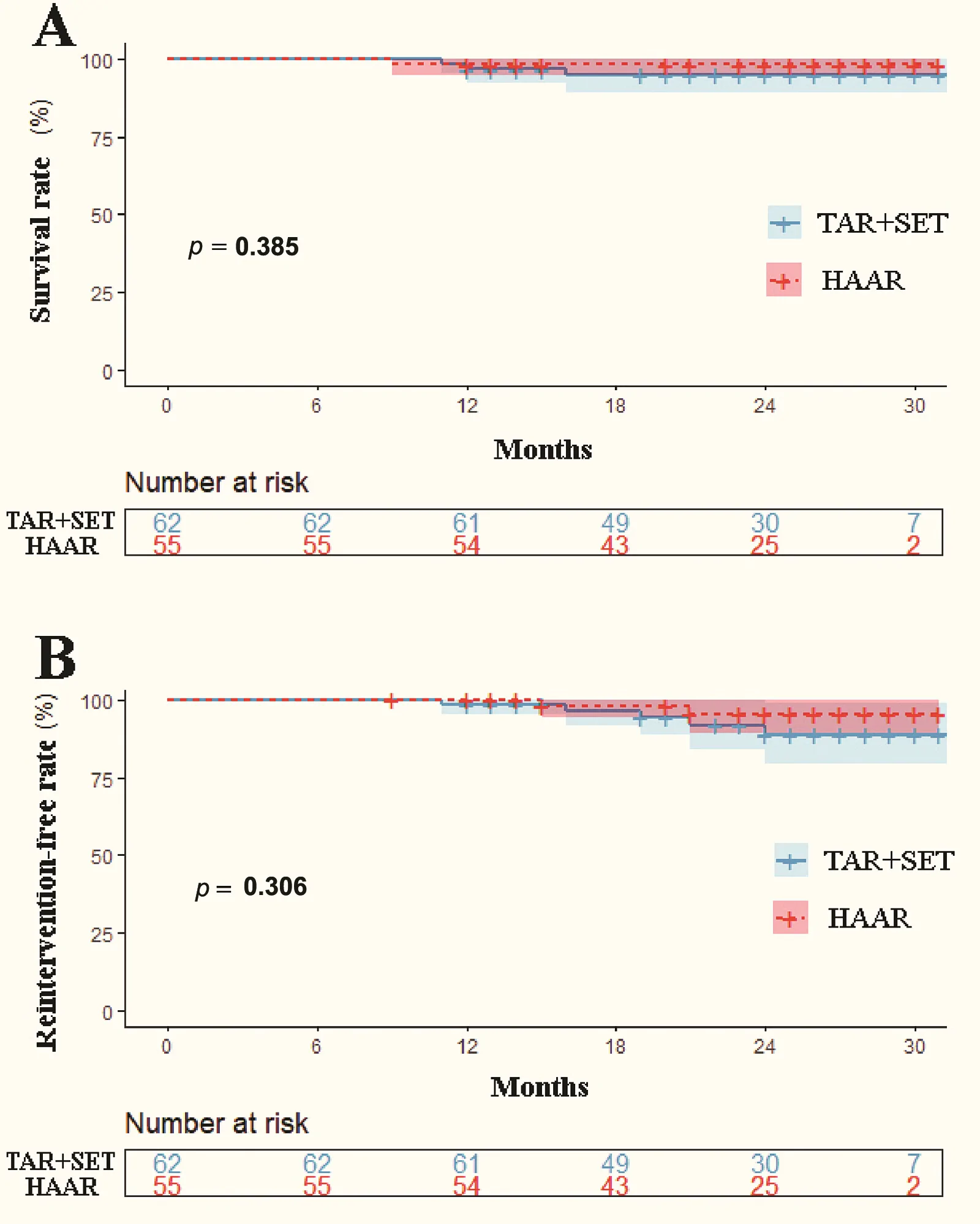
( **A** ) The Kaplan–Meier survival curves according to procedure type. The 2-year survival rate was 94.8% (95% CI, 84.8–98.3%) in the TAR + SET group and 98.2% (95% CI, 87.8–99.7%) in the HAAR group. ( **B** ) Reintervention-free rates according to procedure type. The reintervention-free rate at 2 years was 88.7% (95% CI, 74.3–95.3%) in the TAR + SET group and 95.4% (95% CI, 82.5–98.8%) in the HAAR group. The colored shaded area represents the range of 95% confidence limits (CIs). HAAR, hybrid aortic arch repair; SET, stented elephant trunk; TAR, total arch replacement.

### Follow-Up Imaging Analysis

Complete imaging data were available for 55 patients in the TAR + SET group and for 52 patients in the HAAR group. No detectable migration or endoleak was observed in either group. At the tracheal bifurcation level, the perigraft false lumen had thrombosed in both groups before discharge. The fate of the DTA and abdominal aorta after surgery is demonstrated in Table 4 . At the last follow-up, the rate of thrombosis of the false lumen at the level of the diaphragm was 65.5% in the TAR + SET group and 76.9% in the HAAR group ( *p* = 0.191). At the level of the celiac trunk, thrombosis was detected in 16.4% of the TAR + SET group and in 26.9% of the HAAR group ( *p* = 0.184). The thrombosis of the residual false lumen seemed to reach more distally in the HAAR group ( Fig. 3 ).

**Table 4:** Fate of the descending thoracic aorta and abdominal aortas after surgery in patients at last follow-up

Thrombosis of the false lumen	TAR + SET ( *n* = 55)	HAAR ( *n* = 52)	*p* -Value
Diaphragm level	36 (65.5%)	40 (76.9%)	0.191
Celiac trunk level	9 (16.4%)	14 (26.9%)	0.184

Abbreviations: HAAR, hybrid aortic arch repair; SET, stented elephant trunk; TAR, total arch replacement.

**Fig. 3:**
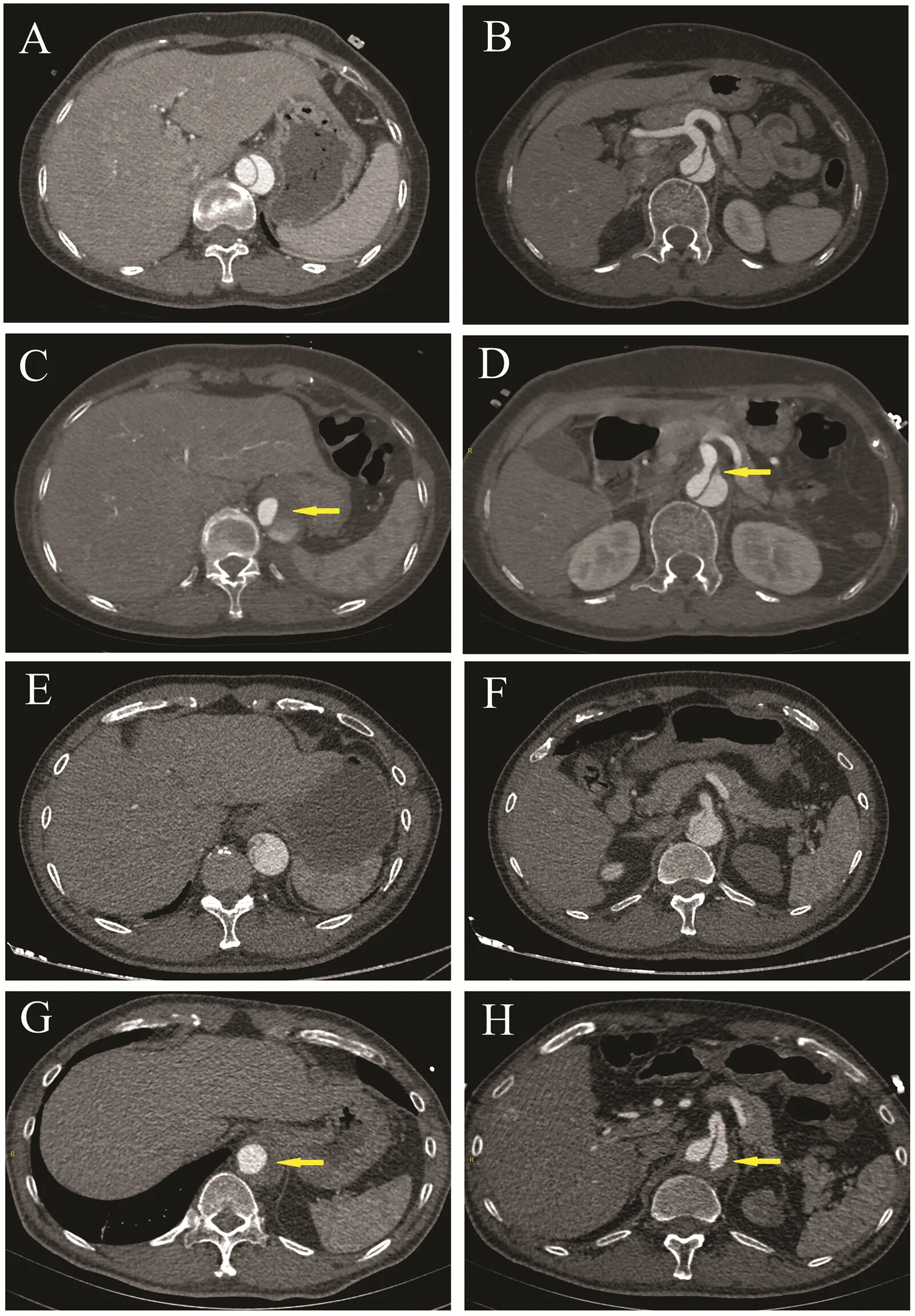
CTA for TAR + SET technique ( **A–D** ) and the HAAR technique ( **E–H** ). The true lumen was compressed by a false lumen before operation: ( **A** ) At the level of the diaphragm in the TAR + SET group; ( **B** ) at the level of the celiac trunk in the TAR + SET group; ( **E** ) at the level of the diaphragm in the HAAR group; ( **F** ) at the level of the celiac trunk in the HAAR group. The extent of thrombosis of the false lumen at 2 years after surgery: ( **C** ) At the level of diaphragm in the TAR + SET group (yellow arrow); ( **D** ) at the level of the celiac trunk in the TAR + SET group (yellow arrow); ( **G** ) at the level of the diaphragm in the HAAR group (yellow arrow); ( **H** ) at the level of the celiac trunk in the HAAR group (yellow arrow). CTA, computed tomography angiography; HAAR, hybrid aortic arch repair; SET, stented elephant trunk; TAR, total arch replacement.

## Discussion

TA-AAD is the most lethal disease of the aorta and has high morbidity and mortality. The primary objective of surgical treatment for TA-AAD is to save the life of patients. 7 Despite the advances in strategic planning, techniques of surgery, anesthesia, organ protection, and perioperative care over the past few decades, mortality rates of aortic dissection remain high. 8 9 10 The traditional surgical management for TA-AAD focuses on handling emergencies in the aortic root and ascending aorta. 11 The remaining untreated distal portion of the aorta has a potential risk of rupture or causing malperfusion syndrome. 12 Owing to the particular anatomic location of branches of the aortic arch, the treatment of TA-AAD always attracted much attention from aortic surgeons. Therefore, an aggressive approach to repair aorta using TAR + SET has been advocated. 13 14 This procedure showed encouraging and promising surgical results. Nevertheless, there were three disadvantages associated with this procedure. First, this procedure still carried the risk of adverse events resulting from DHCA. Second, injury by the distal end of the SET to DTA was related to patients with extremely tortuous morphology of the DTA. Third, the distal end of the SET may enter the false lumen in patients with an entry adjacent to the distal end of the SET, and/or a very small true lumen.

Besides the problems stated above, previous studies reported that the average age of onset of aortic dissection was 60 years, and age was an independent predictor of mortality after TAR surgery. 4 Rudarakanchana and Jenkins reported that TAR surgery remained the gold standard therapy for young patients with TA-AAD. 15 But for patients older than 60 years with TA-AAD, the preferred surgical treatment is still being explored. In recent years, HAAR, which combines surgical and endovascular treatment, was introduced to treat TA-AAD and achieved encouraging results by certain research teams. 16 17 18 19 20

Our current HAAR strategy used in this study can decrease the risk of adverse outcomes, while retaining the advantages of the TAR + SET approach in patients older than 60 years. The results of our analysis demonstrated that HAAR can avoid potential damage from DHCA, which can reduce the use of blood products. The time of aortic cross-clamp, CPB, and surgery duration were reduced in the HAAR group. Moreover, this technique can reduce the time of mechanical ventilation, ICU stay, and hospital length of stay. Early mortality rates and the 2-year survival rates were similar in the HAAR group compared with the TAR + SET group. Furthermore, this technique interfered less with lung and kidney function, compared with the TAR + SET group. This technique significantly reduced the incidence of postoperative pulmonary infection and renal failure. No statistical difference was found between the two groups in the incidence of cardiac dysfunction and hepatic insufficiency.

The association between DHCA and the risk of stroke has been controversial in the literature. 21 22 23 24 25 Retrograde cannulation in the HAAR group may increase the risk of mental dysfunction, compared with antegrade cannulation, because of the higher risk of atheromatous embolization. 26 But, on the basis of our results, it is safe to assume that the HAAR group had a trend toward reducing the rate of stroke and transient mental dysfunction. No paraplegia occurred in either of the groups after the operation. The strategy for complete revascularization of the LSA may have helped to reduce the risk of spinal ischemia even when the endovascular stent graft covered beyond the level of the eighth thoracic vertebrae in the HAAR group.

The HAAR procedure had good intraoperative handling. With respect to the former, our procedure had several advantages. First, the aortic arch was transected between the origin of the innominate artery and the left common carotid artery. This technique prevented injury to the recurrent laryngeal nerve. Second, the proximal landing zone of the stent graft was located in the internal wall of the distal end of the 4-branch prosthetic graft. Retrograde aortic dissection after thoracic endovascular aortic repair was avoided. Third, the stiff guidewire was advanced retrogradely from the femoral artery into the true lumen under fluoroscopy. This avoided the SET from entering the false lumen in the TAR + SET procedure. Fourth, the stent graft was deployed under fluoroscopy. This avoided injury from the stented elephant trunk to DTA. Fifth, the intimal tear in DTA could be sealed off after deployment of the stent graft, where it was reached. In some cases, if one stent graft was inadequate to cover and seal the entry in DTA, a second stent graft can be added. Sixth, stabilization of the distal aortic arch and DTA was also achieved by the stent graft. Seventh, endoleak or incomplete sealing can be examined by angiography. Eighth, visceral malperfusion can be detected by fluoroscopy during the operation. Ninth, it was very easy to achieve hemostasis at the distal anastomosis because the stent graft was deployed in the 4-branch prosthetic graft with an overlapping margin of approximately 2 cm. Tenth, it enlarged the true lumen, reestablished flow in the true lumen, and promoted thrombosis of the distal residual dissected aorta.

Our studies have also shown that the false lumen was obliterated with thrombus at the level of the diaphragm and celiac trunk in more patients in the HAAR group. The need for secondary intervention of the DTA was decreased in the HAAR group, as demonstrated by our follow-up results. Because of the small sample, the results fail to reach statistical significance between the two groups. After remodeling of the distal dissected aortic wall, the DTA and abdominal aorta were stabilized.

## Limitations

1. It was a retrospective and observational study.
2. The number of patients who underwent the HAAR procedure in this study comprises a relatively small sample.
3. The follow-up period was relatively short to evaluate the durability of the endovascular stent grafts and clinical outcomes.

## Conclusion

This study indicated that the HAAR procedure could be safely performed in patients older than 60 years with TA-AAD. In comparison with the TAR + SET procedure, the HAAR procedure may be associated with encouraging surgical results and promising outcomes in the early and mid-term. Furthermore, this procedure could provide a satisfactory remodeling effect of the dissected DTA and abdominal aorta after the operation. Long-term results are required to further validate the present results.
